# Deciphering the genomic character of the multidrug-resistant *Staphylococcus aureus* from Dhaka, Bangladesh

**DOI:** 10.3934/microbiol.2024036

**Published:** 2024-09-29

**Authors:** Afia Anjum, Jarin Tabassum, Sohidul Islam, A. K. M. Imrul Hassan, Ishrat Jabeen, Sabbir R. Shuvo

**Affiliations:** Department of Biochemistry & Microbiology, North South University, Dhaka, Bangladesh

**Keywords:** *S. aureus*, Bangladesh, MRSA, phylogenomic, resistance mechanisms

## Abstract

*Staphylococcus aureus* is one of the leading agents of nosocomial and community-acquired infections. In this study, we explored the genomic characterization of eight methicillin-resistant clinical isolates of *S. aureus* from Dhaka, Bangladesh. Notably, all strains were resistant to penicillin, cephalosporins, and monobactams, with partial susceptibility to meropenem and complete susceptibility to amikacin, vancomycin, and tigecycline antibiotics. The strains were found to have an average genome size of 2.73 Mbp and an average of 32.64% GC content. Multi-locus sequence typing analysis characterized the most predominant sequence type as ST361, which belongs to the clonal complex CC361. All isolates harbored the *mecA* gene, often linked to SCC*mec*_type IV variants. Multidrug resistance was attributed to efflux pumps NorA, NorC, SdrM, and LmrS alongside genes encoding beta-lactamase BlaZ and factors like ErmC and MepA. Additionally, virulence factors including *adsA*, *sdrC*, *cap8D*, *harA*, *esaA*, essC, *isdB*, *geh*, and *lip* were commonly identified. Furthermore, genes associated with heme uptake and clumping were present, highlighting their roles in *S. aureus* colonization and pathogenesis. Nine secondary metabolite biosynthetic gene clusters were found, of which six were common in all the strains. Numerous toxin-antitoxin systems were predicted, with ParE and ParB-like nuclease domains found to be the most prevalent toxin and antitoxin, respectively. Pan-genome analysis revealed 2007 core genes and 229 unique genes in the studied strains. Finally, the phylogenomic analysis showed that most Bangladeshi strains were grouped into two unique clades. This study provides a genomic and comparative insight into the multidrug resistance and pathogenicity of *S. aureus* strains, which will play a crucial role in the future antibiotic stewardship of Bangladesh.

## 1. Introduction

Multidrug-resistant (MDR) *Staphylococcus aureus* is capable of manifesting in a wide range of nosocomial and community-acquired infections, from mild erythema to potentially deadly diseases like endocarditis, pneumonia, and septicemia [1]–[4]. The species can carry a multitude of resistance determinants [5], and it is becoming increasingly difficult to combat them because of resistance to antibiotics like cefotaxime and cefepime, making them clinically significant [6],[7]. Besides, the seven common antibiotics currently used to treat *S. aureus* infections, namely vancomycin, daptomycin, linezolid, sulfamethoxazole and trimethoprim, quinupristin-dalfopristin, clindamycin, and tigecycline are also losing their efficacy [8]–[10]. The emergence of MDR *S. aureus* has led to the development of complex resistance mechanisms, which include chromosomal intrinsic resistance, plasmid, and mobile genetic elements, including staphylococcal cassette chromosome *mec* (SCC*mec*)-mediated acquired resistance and an active efflux system [11]–[13]. Besides, the pathogenicity of this species is predominantly aggressive, as it carries genes encoding a wide range of virulence factors that contribute to its survival, transmission, and nutrient acquisition [14]–[16].

Management of staphylococcal infection has not been extensively monitored in Bangladesh [17], with limited data regarding the prevalence and genotypes of different MDR *S. aureus* strains, including methicillin-resistant *S. aureus* (MRSA). In contrast, many other Asian countries, including Japan, China, and India, have had extensive surveys providing much better insights [18],[19]. The existing data, nevertheless, is alarming, as Bangladesh shows an increasing trend of MRSA in patients ranging from 15.38% to 80.3% [6],[20]–[22]. This aggravating situation makes it imperative for Bangladesh to have a comprehensive understanding of the cause and prevalence of antibiotic resistance.

In this present study, we have explored the genomic characterization of MDR clinical isolates of *S. aureus* from Dhaka, Bangladesh by a genomic study, with a particular focus on genetic variation, antimicrobial resistance, virulence profile, and phylogenomic analysis. To the best of our knowledge, this is one of the first genomic studies on *S. aureus* in Bangladesh.

## 2. Materials and methods

### 2.1. Isolation and phenotypic characterization of *S. aureus* strains

In January 2022, a total of eight *S. aureus* isolates were collected from clinical specimens from Dhaka, Bangladesh. SAC1, SAC3, SAC4, SAC5, and SAC6 were isolated from wound swabs from five inpatients from the burn unit of Dhaka Medical College. Isolates SAC8, SAC9, and SAC10 were obtained from outpatient blood cultures from the LABAID Diagnostic Ltd., Mirpur, Dhaka. Isolation of the *S. aureus* strains was performed on mannitol salt agar (MSA; Condalab, Spain), followed by the subculture on MSA to observe their distinctive characteristics. All strains were stored in Luria-Bertani broth (HiMedia, India) containing 50% glycerol at −80 °C until further use. All procedures were approved by the North South University Research Ethics Committee (IRB: 2022/OR-NSU/IRB/0703).

The confirmed *S. aureus* isolates were tested to identify their antimicrobial resistance pattern by using the Kirby-Bauer disk diffusion technique [23] on Mueller-Hinton agar (HiMedia, India) according to the Clinical and Laboratory Standards Institute (CLSI) [24] guideline (Table S1). The antibiotics tested were ampicillin (AMP, 30 µg), amoxicillin with clavulanic acid (AMC, 30 µg), tazobactam with piperacillin (TZP, 110 µg), ceftazidime (CAZ, 30 µg), cefixime (CFM, 5 µg), ceftriaxone (CRO, 30 µg), cefotaxime (CTX, 30 µg), cefepime (FEP, 5 µg), aztreonam (ATM, 30 µg), meropenem (MEM, 10 µg), amikacin (AMK, 30 µg), gentamicin (GEN, 10 µg), ciprofloxacin (CIP, 5 µg), levofloxacin (LEV, 5 µg), erythromycin (ERY, 15 µg), tetracycline (TET, 30 µg), tigecycline (TGC, 15 µg), and colistin (COL, 10 µg) (Bioanalyse, Turkey). The methicillin resistance was confirmed using cefoxitin (FOX, 30 µg) [25]. The minimum inhibitory concentration (MIC) of *S. aureus* strains against vancomycin (VAN) was determined by broth microdilution method following CLSI guidelines. Briefly, the strains were grown in cation-adjusted Mueller–Hinton broth (Condalab, Madrid, Spain). The overnight bacterial suspensions were adjusted to 0.5 McFarland standard and were grown for 20 h at 37 °C in the presence of vancomycin (Opsonin Pharmaceutical, Bangladesh). The vancomycin concentrations used ranged from 0.5 to 256 µg/mL. Each experiment was performed in duplicates, and bacterial growth was visually observed.

Biofilm formation was determined by a conventional microtiter-plate assay with minor modifications [26]. A single colony from each culture plate was picked and incubated overnight at 37 °C in a shaking incubator at 220 rpm. The inoculum was then diluted 1:1000 in nutrient broth and incubated at 37 °C for 48 h under static conditions. Biofilm formation was assessed using 0.1% crystal violet staining. The biomass was dissolved with 30% acetic acid to quantify the biofilm, and its absorbance was measured at 590 nm using a microplate reader (Multiskan EX, Thermo Scientific, Finland). All procedures were conducted at room temperature and repeated three times under identical conditions.

### 2.2. Genome assembly and annotation

The genomic DNA of the *S. aureus* isolates was extracted using Wizard® Genomic DNA Purification Kit (Promega, USA) following the manufacturer's instructions. The quantity and quality of extracted DNA was determined using a NanoDrop™ 2000 (Thermo Scientific, USA). Sequence raw read files of all *S. aureus* strains were generated using Ion Torrent Sequencing Technology on an Ion GeneStudio™ S5 System (Thermo Fisher Scientific, USA) according to the manufacturer's instructions (DNA Solution Ltd, Dhaka, Bangladesh). For each sample, multiple reads were generated and quality control and adapter trimming were performed using the integrated Torrent Suite™ Software version 5.10.0. Assembly was done using Unicycler version v0.4.8 [27] and SAMtools version 1.11 [28]. To assess the quality of assembly, Quast v5.0.2 was used [29]. The annotations were done using the Rapid Annotation using Subsystem Technology (RAST) tool kit (RASTtk) [30]. The assembly and annotation services were provided by the Bacterial and Viral Bioinformatics Resource Center (BV-BRC) [31],[32]. Proksee was used to generate a circular map reflecting the local alignment of the strains [33]. Assembled draft genomes were stored at the National Center for Biotechnology Information (NCBI) (BioProject: PRJNA983588).

### 2.3. Prediction of antibiotic resistance genes and genomic characterization of MRSA strains

Assembled draft genomes were used in the Resistance Gene Identifier (RGI) tool offered by the Comprehensive Antibiotic Resistance Database (CARD) to predict the resistomes of the strains [34]. The SCC*mec*Finder-1.2 was used to predict the SCC*mec* types [35]–[37]. Multi-locus sequence typing (MLST) of *S. aureus* isolates was determined by MLST-2.0 web server [37]–[39]. MLST was performed by identifying different variants located in seven housekeeping genes, carbamate kinase (*arcC*), shikimate dehydrogenase (*aroE*), glycerol kinase (*glpF*), guanylate kinase (*gmk*), phosphate acetyltransferase (*pta*), triosephosphate isomerase (*tpi*), and acetyl coenzyme A acetyltransferase (*yqiL)*. Clonal complexes (CC) were assigned comparing our MLST data with the PubMLST *S. aureus* typing database (updated August 12, 2024) using the Burst analysis software available on the PubMLST server and were defined as single-locus variants (SLVs) [40]. SpaTyper-1.0 [38],[41], alongside basic local alignment search tool (BLAST) from NCBI, were used for staphylococcal protein A (spa) typing. Pathogenfinder-1.1 [42] web service was offered by the Center for Genomic Epidemiology (CGE) [37],[43] and used to determine the pathogenicity of the strains. Default parameters were used for all the services.

### 2.4. Prediction of mobilome, virulome, secondary metabolite cluster, and toxin-antitoxin system

Plasmidfinder-2.1 [37],[43], Phage Search Tool Enhanced Release (PHASTER) [44],[45], and ICEfinder-1.0 [46] for Integrative and Conjugative Elements (ICE's) were used to identify plasmid, phage and type IV secretion system of the strains, respectively. Default parameters were used for all programs. The presence of virulence factors for the strains was predicted using the Virulence Factor Database (VFDB) (http://www.mgc.ac.cn/VFs/) [47]. The strains were systematically scanned by Antibiotics & Secondary Metabolite Analysis Shell (antiSMASH) v7.0.1 with default parameters for the number and types of secondary metabolite Biosynthetic Gene Clusters (BGCs) present [48]. Toxin-antitoxin systems mania (TASmania) [49] was used to predict toxin-antitoxin systems.

### 2.5. Comparative analysis

The Bacterial Pan Genome Analysis (BPGA) v1.3 [50] tool was used for pan-genome analysis. Average nucleotide identity (ANI) analysis was performed using Kostas Lab [51] (Figure S1). A phylogenomic tree was constructed from fifty closely related *S. aureus* strains isolated worldwide based on ANI (Figure S1 and Table S2). Phylogenomic analysis was carried out in the Type Strain Genome Server (TYGS) (https://tygs.dsmz.de) for a whole genome-based taxonomic analysis [52]. Later, CSIPhylogeny version 1.4 was used for the generation of a phylogenomic tree against the reference genome *S. aureus* NCTC 8325 (accession number: CP000253) based on single nucleotide polymorphism (SNP) [53]. Both the phylogenomic trees were visualized by the Interactive Tree of Life (iTOL) [54]. All heatmaps were generated using Science and Research (SR) online Plot (www.bioinformatics.com.cn).

## 3. Results and discussion

### 3.1. Antibiotic resistance profile and characterization of the Bangladeshi *S. aureus* isolates

**Figure 1.**
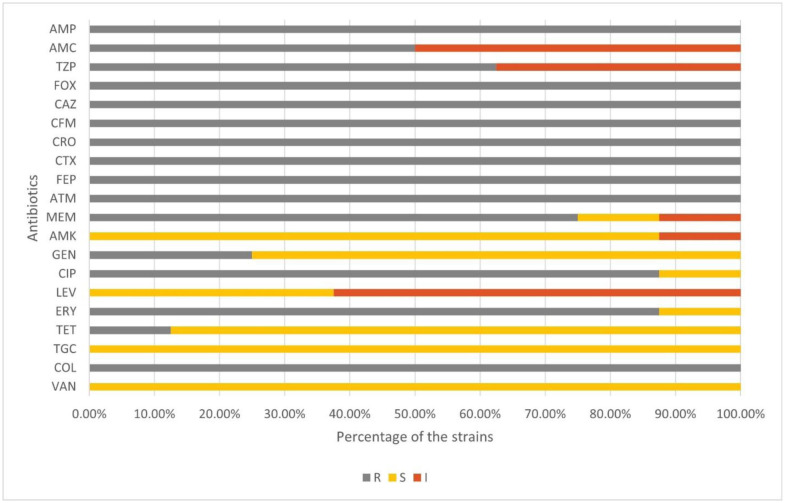
Antibiotic resistance profile of the *S. aureus* clinical isolates.

An increasing incidence of MDR *S. aureus* (97.4%) infections has recently been reported in Bangladesh [55]. Among the twenty antibiotics used in this study, all isolated strains demonstrated resistance to eight, namely ampicillin, ceftazidime, cefixime, ceftriaxone, cefotaxime, cefepime, aztreonam, and cefoxitin, as shown in Table 1 and Figure 1. The MIC of vancomycin for all the strains was found to be 1 µg/mL using the broth microdilution method, which is considered sensitive according to CLSI guidelines. However, *S. aureus* is intrinsically resistant to colistin, which was also observed in this study. Therefore, tigecycline, vancomycin, and amikacin antibiotics were the most effective against all strains, whereas only SAC 4 showed intermediate resistance to amikacin. In addition, weak biofilm formation was observed in *S. aureus* strains SAC 1, 3, 5, 6, and 8, whereas SAC 4, 9, and 10 were non-biofilm formers (Table S3).

### 3.2. Genomic features of the isolated *S. aureus* strains

**Figure 2.**
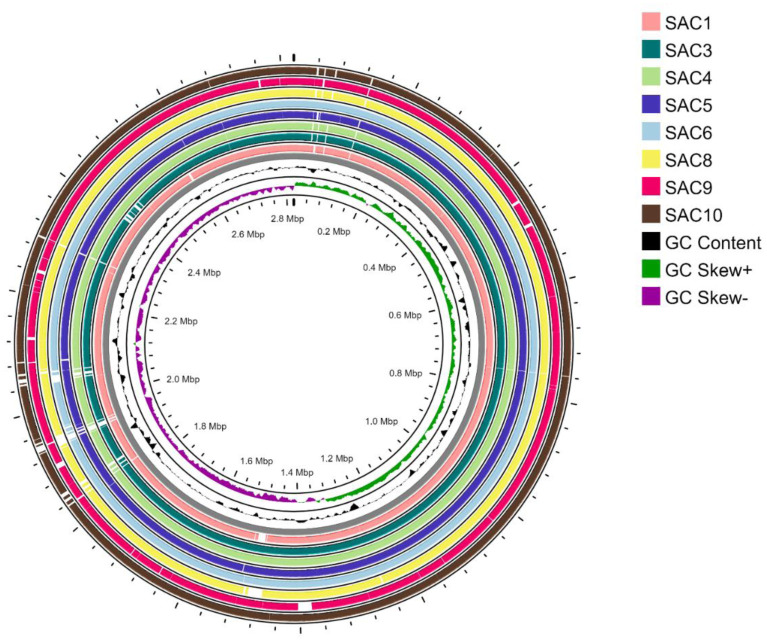
Sequence alignment of the isolated *S. aureus* strains. The gaps on each circular genome represent the missing regions identified in BLAST analysis. The inner circle represents the sequence clockwise. The GC content is shown in black. The positive GC skew is shown in green, and the negative GC skew is visualized in purple.

The studied isolates were found to have an average genome size of 2.73 Mbp. SAC 6 had the largest genome size at 2.84 Mbp (Table 2 and Figure 2). The isolates had an average of 32.64% GC content and an average of 2698.5 coding sequences (CDs). SAC 4 had the maximum predicted coding sequence of 2963. The draft genome sequences were aligned with reference genome NCTC 8325 (Figure 2), representing the gaps in the draft genomes. The subsystem superclass distribution identified ‘metabolism’ as the most predominant subsystem for all the strains (Figure S2).

**Table 1.** Antibiotic sensitivity pattern of the studied *S. aureus* clinical isolates.

Antibiotic class	Penicillin	Penicillin + beta-lactamase inhibitors		Cephalosporin						Monobactam	Carbapenem	Aminoglycoside		Fluoroquinolone		Macrolide	Tetracycline		Polymyxin	Glycopeptide
Strains (SAC)	AMP	AMC	TZP	FOX	CAZ	CFM	CRO	CTX	FEP	ATM	MEM	AMK	GEN	CIP	LEV	ERY	TET	TGC	COL	VAN
SAC1	R	I	R	R	R	R	R	R	R	R	S	S	S	R	I	R	S	S	R	S
SAC3	R	I	I	R	R	R	R	R	R	R	R	S	S	R	S	R	R	S	R	S
SAC4	R	I	I	R	R	R	R	R	R	R	I	I	S	R	I	R	S	S	R	S
SAC5	R	I	I	R	R	R	R	R	R	R	R	S	S	S	S	S	S	S	R	S
SAC6	R	R	R	R	R	R	R	R	R	R	R	S	R	R	S	R	S	S	R	S
SAC8	R	R	R	R	R	R	R	R	R	R	R	S	S	R	I	R	S	S	R	S
SAC9	R	R	R	R	R	R	R	R	R	R	R	S	R	R	I	R	S	S	R	S
SAC10	R	R	R	R	R	R	R	R	R	R	R	S	S	R	I	R	S	S	R	S

*R = resistant; S = sensitive; I = intermediate

The MRSA isolates of our study were assigned to 4 genetic lines (CC5, CC8, CC80, and CC361) with their sequence types matching the central genotype at ≥ 6 loci. From the MLST analysis, it was found that SAC 5 and 9 belong to sequence type ST6 (CC5), prevalent in Bangladesh [56]. The MLSTs (other than ST6) identified in the isolates appear to be unique or not previously registered in clinical isolates of Bangladesh according to the PubMLST database [40]. SAC1 was found to belong to ST80 (CC80), which is considered one of the most important toxinogenic clones present in the species across the world [57]. 50% of the isolated strains were classified as part of the MLST type 361 (Table 2). Despite the apparent bias of the study with the small number of strains used, the results are consistent with established knowledge of MRSA isolates through literature [58]–[61]. In multiple studies, including one in Bangladesh, the comparative sporadicity of ST361 was reported [62],[63], which demonstrated a gradual change with more cases linked to ST361 (CC361) [58]. Subsequent documentation revealed the presence of ST361 with the SpaType T315 in the processed fish fingers and Chatpatti in Dhaka, Bangladesh [59]; similar Sequence and Spa Type were recovered from patients in Irish hospitals between 2000 and 2012 [60] and in Kuwait in 2010 [61]. The rest of the isolates (SAC 3, 4, 8) have different SpaTypes (Table 2).

**Table 2.** Genomic features of the *S. aureus* clinal isolates.

Name of the strains	Genome size (bp)	aN50	bL50	GC (%)	Coding genes	tRNA & rRNA	MLST	Clonal complex (CC)	Spa types	NCBI BioSample ID
SAC 1	2,787,226	176,673	7	32.71	2715	61	ST80	CC80	T376	SAMN35731402
SAC 3	2,754,801	131,013	5	32.66	2721	57	ST361	CC361	T304	SAMN35731599
SAC 4	2,814,822	116,081	7	32.64	2963	57	ST361	CC361	T463	SAMN35731600
SAC 5	2,784,621	111,616	8	32.69	2714	22	ST6	CC5	T4407	SAMN35731637
SAC 6	2,846,622	141,025	8	32.58	2807	59	ST8	CC8	T3364	SAMN35731648
SAC 8	2,709,879	70,277	14	32.62	2616	21	ST361	CC361	T2379	SAMN35731650
SAC 9	2,817,774	192,408	7	32.71	2752	58	ST6	CC5	T304	SAMN35731660
SAC 10	2,801,390	108,408	8	32.65	2741	59	ST361	CC361	T315	SAMN35731667

**^a^N50 = Half of the genome assembly is contained in contigs equal to or larger than this value; ^b^L50 = smallest number of contigs (each with its length) in the genome assembly needed to cover approximately half of the total genome size.

The strains were subjected to prophage sequence identification, leading to the determination of two intact prophage regions in SAC 1, 3, 4, 5, and 9 (Table S4). SAC 8 and 10 have only one predicted prophage in their genomes. Except for SAC 8, which contains a single plasmid, the rest of the strains harbor multiple plasmids ranging from 2 to 6. Moreover, SAC 1, 3, 8, and 10 had putative integrative and conjugative element (ICE) regions with T4SS (Table S4). Pathogen finders predicted (97%–98%) that all the strains were to be human pathogens.

### 3.3. Antibiotic resistance of the isolated *S. aureus* strains

**Figure 3.**
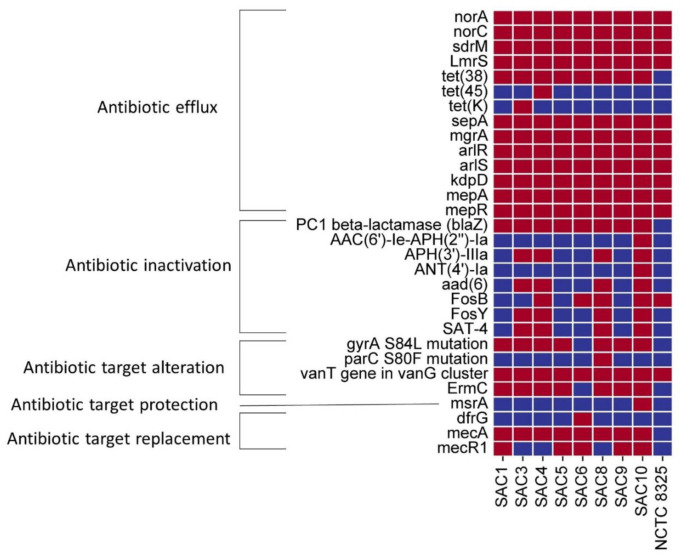
Antibiotic resistance genes of all the isolated Bangladeshi *S. aureus* strains and the reference NCTC 8325. The red squares denote the presence of the genes, and the blue squares denote the absence of the genes listed.

Several antibiotic-resistant genes, ranging from efflux pumps to antibiotic inactivation, antibiotic target alteration, protection, and replacement, have been predicted by CARD in the studied *S. aureus* clinical strains from Bangladesh. All strains studied were found to be MRSA and predicted to have *mecA* (Table 1 and Figure 3), conferring the presence of SCC*mec* genomic islands. However, *mecR1*, a gene that encodes a membrane-spanning signal transduction protein responsible for the upregulation of *mecA*, is only present in SAC 1, 5, 6, 9, 10. These five strains also contained variations of SCC*mec* type_IV, while SCC*mec*_type_IVa (2B) was the most common (Table 3). SAC 1, 5, and 9 had SCC*mec* subtype a, whereas SAC 6 and 10 contained subtypes c and g, respectively. The strains with identifiable SCC*mec* elements contained ccrA2/ccrB2 recombinases (Table S5). The server was unable to detect a complete SCC*mec* element in SAC 3, 4, and 8 with a template coverage of at least 40% but was found to carry *ccrC1*-allele-2. Besides, in these strains, MecR1 was also absent (Figure 3). SAC 8 was found to have the class complex *mec*-class-C2 (Table S5), which is composed of insertion sequence IS431, truncated remnants of *mecR1*, *mecA*, and another copy of IS431 in the opposite direction. Based on the combination of the *mec* complex class and the *ccr* complex type, SAC 8 could belong to SCC*mec*_type_V [64].

**Table 3.** SCC*mec* types and predicted *ccr* gene.

Strains	SCC*mec* types	Predicted *ccr* gene
SAC 1	SCC*mec*_type_IVa(2B)	*ccrA2, ccrB2*
SAC 3	Not detected	*ccrC1*-allele-2
SAC 4	Not detected	*ccrC1*-allele-2
SAC 5	SCC*mec*_type_IVa(2B)	*ccrA2, ccrB2*
SAC 6	SCC*mec*_type_IVc(2B)	*ccrA2, ccrB2*
SAC 8	Not detected	*ccrC1*-allele-2
SAC 9	SCC*mec*_type_IVa(2B)	*ccrA2, ccrB2*
SAC 10	SCC*mec*_type_IVg(2B)	*ccrA2, ccrB2*

In this study, 100% of the studied *S. aureus* were found to be resistant to penicillin, cephalosporin, and monobactam class of antibiotics (Figure 1). Only two out of eight MRSA strains were either susceptible or intermediately susceptible to meropenem. The efficacy of penicillin, either amoxicillin or tazobactam, increased when tested along with beta-lactamase inhibitors (Table 1). The beta-lactamase inhibitors improved the susceptibility test results from resistant to intermediately resistant only in 40%–50% of the strains when clavulanic acid was used with amoxicillin or piperacillin was used with tazobactam. All these strains harbored PC1 beta-lactamase *blaZ* (Figure 3).

Aminoglycosides appear to be relatively successful in neutralization of the clinical strains, where only 1 out of the 8 strains showed intermediate resistance to amikacin, and two strains showed resistance to gentamicin (Table 1). The genes that are typically responsible for encoding the modifying enzymes conferring aminoglycoside resistance through antibiotic inactivation include *AAC(6′)-APH(2")-la, APH(3′)-IIIa, ANT(4′)-Ia*, and *aad (6)* [65],[66], each of which was detected only in SAC 10. However, SAC 10 was found to be sensitive to both the aminoglycosides amikacin and gentamicin. *APH (3′)-IIIa*, conferring resistance to amikacin [67], along with *aad(6)*, were also detected in SAC 3, 4, 8, and 10. Conversely, only SAC 4 showed intermediate resistance to amikacin. The strains SAC 6 and 9, despite the absence of all of these aminoglycoside modifying enzymes (AMEs), showed resistance to gentamicin. Given the limited detection of AME-encoded genes and the lack of a clear phenotypic correlation with their presence, alternative resistance mechanisms might be responsible for the observed aminoglycoside resistance in these two isolates.

The studied *S. aureus* strains harbored genes for various superfamilies of efflux pumps. Notably, NorA, NorC, and SDrM are described as the cause of fluoroquinolone antibiotic resistance in *S. aureus* [68]–[70]. All but strain SAC 5 were resistant to the second-generation fluoroquinolone ciprofloxacin, and none of the strains were resistant to the third-generation fluoroquinolone levofloxacin. The efflux pump encoding genes *norA*, *norC*, and *sdrM* conferring resistance to fluoroquinolones were predicted in every strain, including the reference strain NCTC 8325. While the presence of genes encoding efflux pumps suggests a potential resistance mechanism for fluoroquinolones, the expression of these pumps likely requires prior exposure to fluoroquinolones. SAC 5, a clinical strain, may have undergone antibiotic exposure other than fluoroquinolones and thus developed resistance to those antibiotics.

A previous study demonstrated a biphasic killing response of the reference strain *S. aureus* NCTC 8325 to ciprofloxacin. At concentrations ≥ 5 µg/mL, NCTC 8325 exhibits persister formation [71]. However, this strain remains susceptible at lower concentrations (1 µg/mL) despite harboring genes predicted to encode efflux pumps [71]. This observed difference in susceptibility compared to clinical isolates, which may have undergone selective pressure leading to efflux pump overexpression, suggests that these genes alone may not be sufficient for ciprofloxacin resistance in NCTC 8325.

The contribution of the efflux pump and their elevated expression during antibiotic pressure in clinical settings, along with quinolone resistance determining region (QRDR) mutations, may contribute to the fluoroquinolone resistance of these strains. Among the QRDR mutations, the *parC* gene, predicted to carry a single nucleotide polymorphism S80F (Table S6), and the *gyrA* gene, predicted to carry S84L mutation, are known to confer resistance to fluoroquinolones [72],[73], both of which were predicted in SAC 8. This may explain why SAC 8 exhibits complete or intermediate resistance to ciprofloxacin and levofloxacin, respectively (Table 1, Figure 1). SAC 1, 4, 9, and 10 also showed the same resistance pattern concerning fluoroquinolones as SAC 8 despite lacking the *parC* S80F mutation in the respective strains. The efflux pumps and the *gyrA* mutation may contribute to fluoroquinolone resistance. SAC 6, notably missing the *gyrA* mutation, was found to be resistant to ciprofloxacin yet sensitive to levofloxacin, thus attributing its ciprofloxacin resistance to the efflux pumps.

The MFS efflux pump LmrS was also detected in all Bangladeshi-resistant strains and can efflux several structurally unrelated drugs, which include lincomycin, kanamycin, fusidic acid, etc. [68]. These strains also contained multidrug and toxic compound extrusion (MATE) superfamily efflux transporter gene *mepA* and small multidrug resistance (SMR) superfamily efflux transporter SepA. MepA confers resistance to tetracycline and contributes to decreased susceptibility to tigecycline antibiotics [74],[75], whereas SepA is reported to efflux disinfecting agents and antiseptics [76].

A recent study on resistance to macrolide antibiotics of *S. aureus* strains confirmed the *ermC* gene to be the most common determinant of macrolides, lincosamides, and streptogramin B (MLS_B_) resistance, compared to *msrA* [77]. Our study consistently predicted the presence of the *ermC* gene in each of the clinical strains except SAC 6, which confer erythromycin resistance by methylating 23S rRNA [78]. Only SAC 10 was predicted to carry the *msrA* gene encoding the ATP-dependent efflux pump (ABC), conferring resistance to certain macrolides and streptogramin type B in *Staphylococcus* spp [79]. However, despite the absence of both *ermC* and *msrA* genes, SAC 6 was resistant to the tested macrolide erythromycin. The efflux pump encoding LmrS predicted in every strain could be the contributing factor since its role in multi-drug resistance including macrolides is well-established [68].

The *tet(K)* gene is one of the major genes associated with tetracycline resistance among Gram-positive bacteria [80]. Among all the strains, only SAC 3 harbored *tet(K)* and showed resistance against this antibiotic. While both *tet(45)* (predicted only in SAC 4) and *tet(38)* (predicted in all strains) encode tetracycline efflux pumps [81],[82], their presence did not correlate with resistance in our study. It is important to note that overexpression is often required for tet(38) and *tet(K)*-like efflux pumps to confer tetracycline resistance [75],[83]. Additionally, *S. aureus* is intrinsically resistant to polymyxins [84]; each of our studied strains was found resistant to colistin.

Finally, some genes were predicted whose associated antibiotics were not tested on our strains including *sepA*, *fosB*, *fosY*, *sat-4*, and *dfrG*. Among the two Fosfomycin-resistance enzymes predicted, *fosB* and *fosY* [85],[86], the latter is a relatively novel member of the Fos family and has profound implications considering its predicted detection in 50% of our strains alongside fosfomycin being a treatment of choice in many cases [86]. The *dfrG* gene (predicted only in SAC 6) is validated to confer trimethoprim resistance [87].

### 3.4. Virulence factors

**Figure 4.**
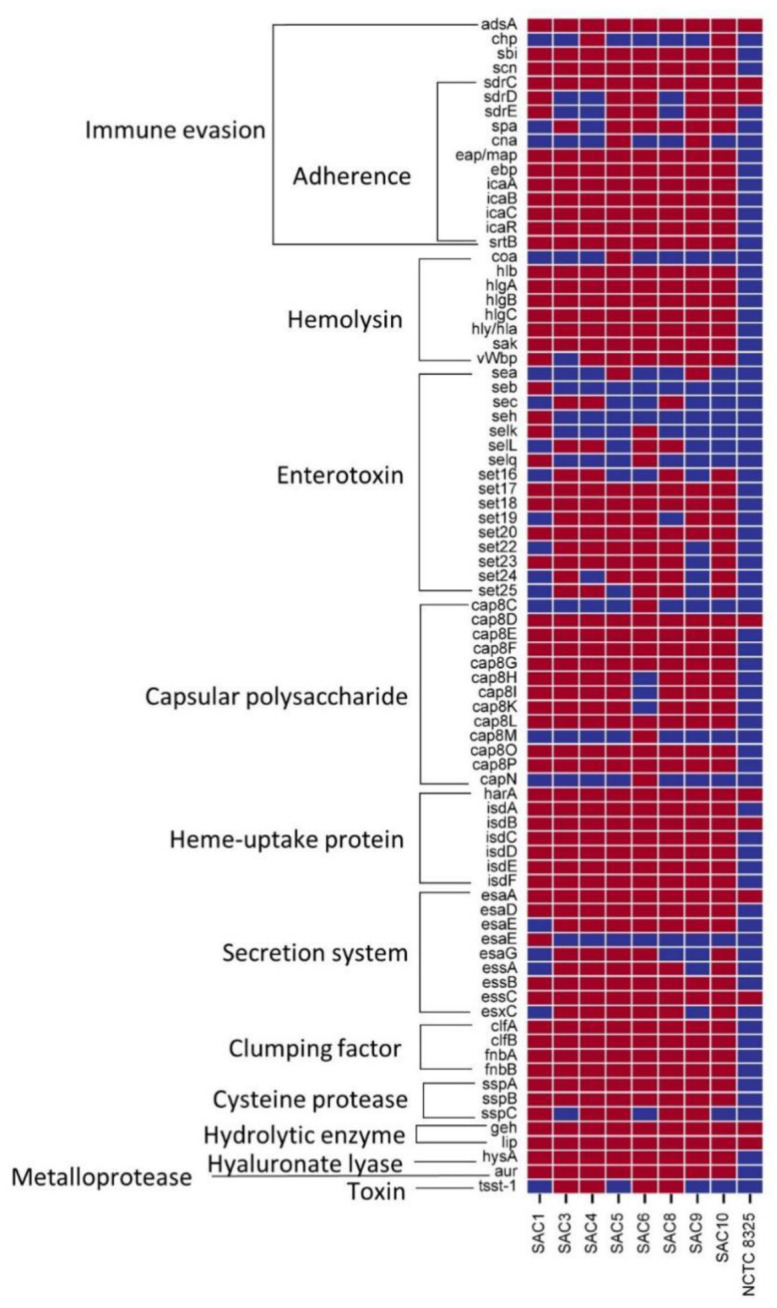
Virulence factors of all the isolated Bangladeshi *S. aureus* strains and the NCTC 8325. The red squares denote the presence of the genes, and the blue squares denote the absence of the genes listed.

Numerous classes of genes for virulence factors were widely distributed among the Bangladeshi *S. aureus* clinical strains, which were completely absent from the reference strain. However, *adsA, sdrC, cap8D, harA, esaA, essC, isdB, geh*, and *lip* were present in each of the strains including the reference NCTC8325 (Figure 4). Most of the virulence factors predicted are responsible for either immune evasion or function as enterotoxins.

In the isolated strains, the virulence genes associated with heme-uptake and clumping were predicted ubiquitously in the isolates, reflecting their significance (Figure 4). The Isd system works together for hemoglobin binding and heme-iron acquisition and is required for the colonization of the host and pathogenesis [88]. IsdB removes heme from bound hemoglobin and transfers this cofactor to other proteins of the Isd system, which import and degrade heme to release iron in the cytoplasm. Apart from differences with respect to the virulence factors like *esxC*, *esaG*, and *essA*, SAC 5 and 9 were the only strains found to carry the gene for the virulence factor *cna*, collagen binding protein. Cna allows both SAC 5 and 9 to adhere to the host and bypass the host immune system, which piques interest as MLST characterized both the isolates in sequence type ST6. This result is consistent with the findings of another study conducted using *S. aureus* strains in India, which established that the strains having the same sequence type tend to follow the same patterns of distribution of virulence factors and immune evasion factors [89]. Drawing further comparison, the virulence factors *spa*, *set24* are missing in SAC 4 but present in SAC 3, whereas the virulence factors *vWbp*, *ssPC*, and *chp* are present in SAC 4, yet missing in SAC 3. Moreover, SAC 3 demonstrates levofloxacin sensitivity and meropenem resistance (Table 1), while SAC 4 exhibits intermediate resistance to both. This suggests that the differential virulence genes pattern may also be responsible for the modest difference in the phenotypic antibiotic resistance pattern [90].

### 3.5. Secondary metabolite cluster

**Figure 5.**
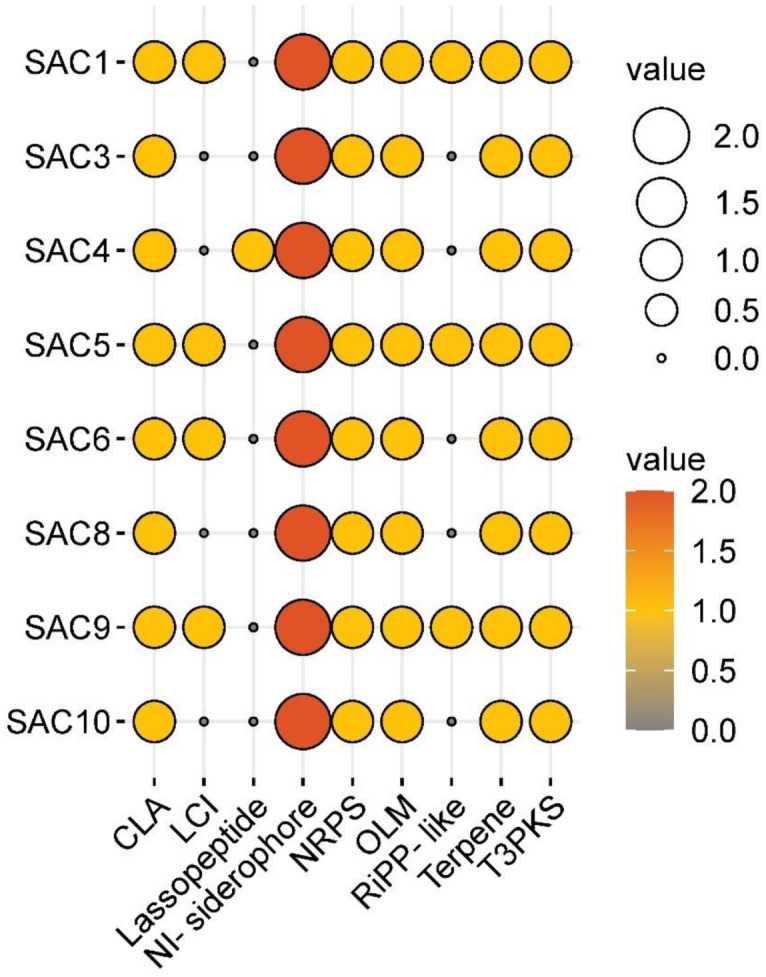
Secondary metabolite biosynthetic gene clusters (BGCs) of the strains. Color intensity and circle size represent the number of gene clusters. Grey and smallest circles represent the absence of gene clusters; yellow and medium circles represent the presence of a single cluster; orange and larger circles represent a higher number of gene clusters. CLA–cyclic lactone autoinducer; LCI–lanthipeptide class I; NI-siderophore - NRPS-independent, IucA/IucC-like siderophores; NRPS–non-ribosomal peptide synthase; OLM–opine-like metallophore; Ripp-like–ribosomally synthesized and post-translationally modified peptide-like.

Secondary metabolites are not crucial for bacterial growth but play various roles in ensuring survival in natural environments [91]. Thus, the biosynthetic gene clusters could provide insight into potential targets to reduce the pathogenicity of the bacteria, as evidenced in numerous studies [92]–[95]. Nine types of secondary metabolite biosynthetic gene clusters (BGCs) were found (Figure 5). Six of the BGCs were present in each of the strains investigated: cylic-lactone autoinducer, opine-like-metallophore, non-ribosomal peptide synthase (NRPS), terpene, and type III Polyketide synthase (T3PKS). T3PKS was mostly found to be located in the same region as that of terpene. However, only SAC 5 was predicted to have the two genes in different regions based on antiSMASH results. Each strain was found to have two non-ribosomal peptide synthetase (NRPS)-independent IucA/IucC like (NI)-siderophore, with the most known cluster corresponding to either staphylofferin A or staphyloferrin B.

The immune effector calprotectin can bind zinc with very high affinity, sequestering zinc away from the pathogen, which provides a layer of nutritional immunity [96]. However, with the metallophore staphylopine, *S. aureus* can compete with the host for zinc, enhancing its ability to cause a successful infection [96]. The (NRPS)-independent IucA/IucC like (NI)-siderophores (most similar with clusters staphyloferrin A and staphyloferrin B) also function in a similar fashion but chelate iron instead of zinc [97]. Lanthipeptide-class-i type was present in four (SAC 1, 5, 6, and 9) of the strains. SAC 1, 5, and 9 were found to carry unspecified ribosomally synthesized and post-translationally modified peptide product (RiPP)-like BGC, whereas lasso peptide was found only in SAC 4. T3PKS lacks investigations with a link to potentially enhanced virulence for *S. aureus*. It is believed that T3PKS may contribute to the persistence of mycobacterium infections through dynamic cell wall remodeling, despite the process not being well understood [98]. The possibility of the same process happening in *S. aureus* requires further research. However, BGCs of type NRPS, with 100% similarity with the known clusters aureusimine A/aureusimine B/aureusimine C are established not to be pathogenic [99]. Class I lanthipeptides, (RiPP)-like BGCs, and lasso peptides detected in the isolates are all ribosomally synthesized and post-translationally modified peptides (RiPPs), showing promise as natural therapeutic alternatives of antibiotics [100]–[102].

### 3.6. Toxin-antitoxin (TA) identification

**Figure 6.**
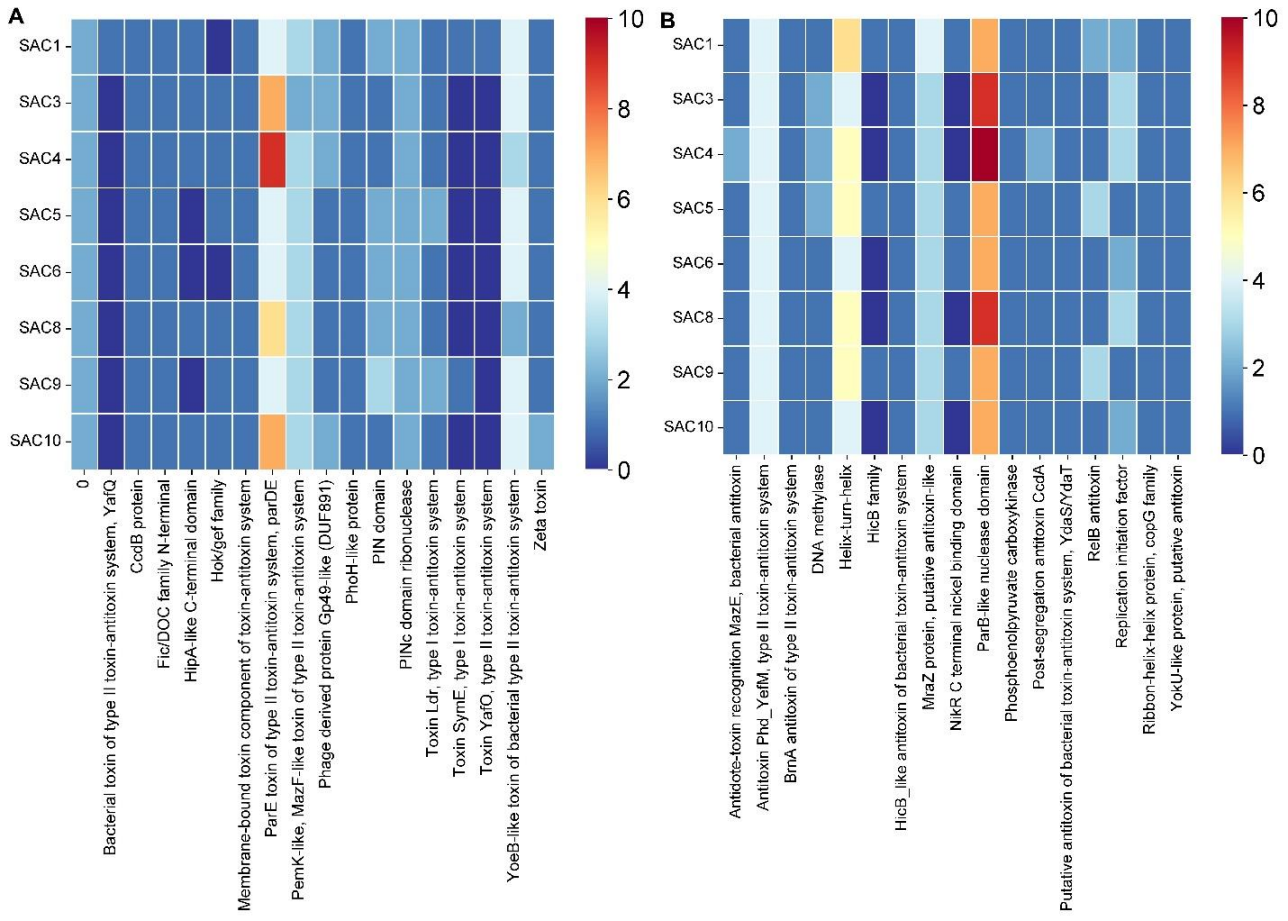
Toxin-antitoxin system of the isolated strains. Different colors represent different numbers of toxin genes (A). Different colors represent different numbers of antitoxin genes (B). The name of each of the strains is listed on the left. The toxin and antitoxins identified are shown at the bottom.

TA systems were analyzed through toxin-antitoxin system mania (TASmania) (Figure 6). It revealed the ParE toxin of the type II TA, ParDE (as the most abundant toxin), with SAC 4 predicted to have nine of them (Figure 6A). An abundance of YoeB-like toxin of bacterial type II TA system was also seen among all the strains. However, Toxin YafO of type II TA system was only carried by SAC 1, whereas Toxin SymE, type I TA system, was carried only by SAC 1 and 9.

The significance of the ParB-like nuclease domain as the most abundant antitoxin was ascertained (Figure 6B). SAC 4 was found to carry ten of the ParB-like nuclease domains as the most abundant antitoxin, whereas SAC 3 and 8 were found to carry eight of each. The most abundant toxin among the strains was ParE of the type II toxin-antitoxin system (Figure 6A), ParDE, which prevents the loss of antibiotic resistance by providing plasmid stability, eliminating the plasmid-free cells [103]. Usually, persister formation is linked to toxin-antitoxin modules only in the case of Gram-negative bacteria like *Escherichia coli* [104],[105]. Besides, antitoxins usually counteract the toxicity of toxins, rendering their functions futile under normal physiological conditions [106]. Antitoxins are unstable as they are continuously degraded and replenished in the Type II TA systems. However, during environmental stress (such as the application of antibiotics), replenishment of antitoxins is not sufficient, and toxins prevail in the toxin-antitoxin ratios [106],[107]. Our study reflects that it is imperative to put more focus on the investigation of TA modules in the case of *S. aureus*. Zeta toxin, part of the Omega/Epsilon/Zeta three-component TA system, is considered the cognate of epsilon antitoxin in existing literature [108]. Though each strain showed hits for the presence of Zeta toxin, not a single instance of hits for Epsilon antitoxin was found (Figure 6B).

### 3.7. Pan-genome analysis

The pan-genome of the eight *S. aureus* isolates characterized in this study had 20,217 genes. It was estimated that the number of core genes shared by all the strains is 2007, whereas the number of accessory genes is 3932. Moreover, there were 229 unique genes in the isolates, where SAC 6, 1, and 10 were found to have acquired the highest number of unique genes, that is, 120, 52, and 26, respectively.

**Figure 7.**
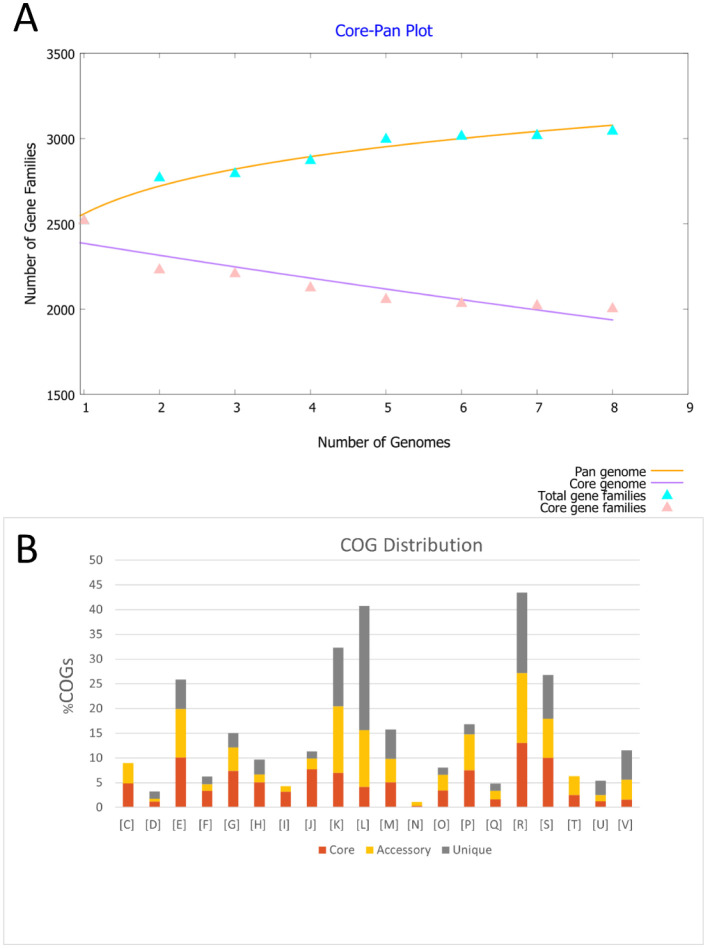
Pan-genome analysis of the isolated *S. aureus* strains. [A] Core–pan plot of the eight *S. aureus* strains studied. [B] Cluster of orthologs groups (COG) distribution of the studied *S. aureus* strains. [C]: Energy production and conversion; [D]: Cell cycle control, cell division, chromosome partitioning; [E]: Amino acid transport and metabolism; [F]: Nucleotide transport and metabolism; [G]: Carbohydrate transport and metabolism; [H]: Coenzyme transport and metabolism; [I]: Lipid transport and metabolism; [J]: Translation, ribosomal structure, and biogenesis; [K]: Transcription; [L]: Replication, recombination, and repair; [M]: Cell wall/membrane/envelope biogenesis; [N]: Cell motility; [O]: Post-translational modification, protein turnover, and chaperones; [P]: Inorganic ion transport and metabolism; [Q]: Secondary metabolites biosynthesis, transport, and catabolism; [R]: General function prediction only; [S]: Function unknown; [T]: Signal transduction mechanisms; [U]: Intracellular trafficking, secretion, and vesicular transport; [V]: Defense mechanisms.

The core–pan plot (Figure 7A) represented that the pan-genome of the clinical *S. aureus* strains was “open” but soon to be closed as the trend curve almost reached a plateau with the addition of more genomes to the analysis. The functional adaptations of Bangladeshi *S. aureus* showed *B*pan values (total expansion rate) of 0.0888959 (i.e., <1) for the number of gene families f(x) in the power-law equation f(x) = a.x^b^, also suggesting that the pan-genome may be closed soon [109],[110]. This shows that the addition of newer genome sequences is unlikely to make a big difference to the pan-genome size, validating that the sequencing effort for MDR *S. aureus* was adequate. The core genome was considered “conserved” since its trendline leveled out.

The cluster of orthologs groups (COG) distribution (Figure 7B) reveals that the genes under the categories of different membrane biogenesis, defense mechanisms, replication, recombination, and repair are mostly unique. Despite having representation in core genomes, the percentage difference highlights the inter-species variations of these categories. Besides, the increasing multidrug resistance can be attributed to the enhanced defense mechanisms or mutations introduced during replication, recombination, and repair, each associated with unique genes. Thus, these categories also reflect the possible role of the addition of unique genes in the genome evolution of the strains in their development of multidrug resistance.

The COG distribution (Figure 7B) predicted the core genomes of the eight investigated strains to be predominantly (about 37%) associated with functions of metabolism and transport of carbohydrates, amino acids, nucleotides, coenzymes, lipids, and inorganic ions. These categories also had a decent representation (about 26%) of accessory genomes. However, the unique genes predominate in categories of cell wall/membrane/envelope biogenesis, defense mechanisms, replication, recombination and repair, and general functions.

**Figure 8.**
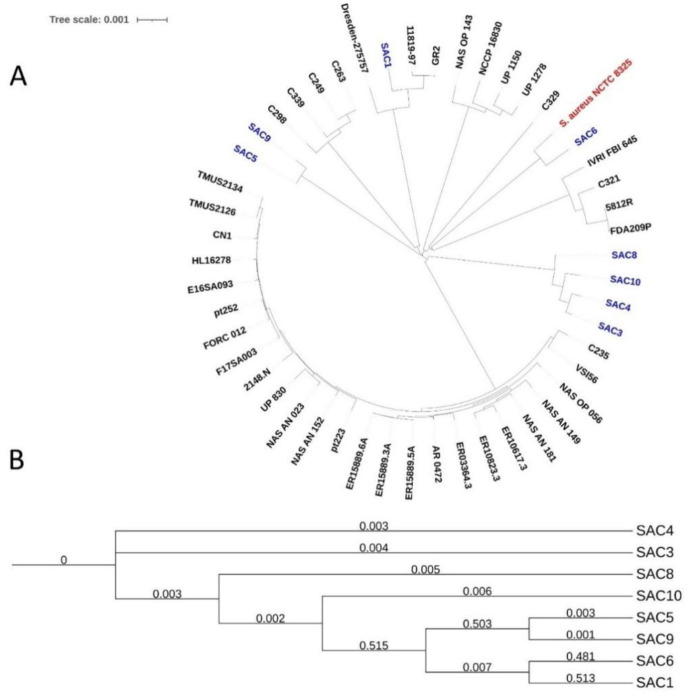
Phylogenomic study of the isolated *S. aureus* strains. (A) Phylogenomic tree of 50 *S. aureus* strains, including 8 Bangladeshi strains, 41 strains isolated worldwide, and reference strain NCTC8325. (B) Phylogenomic tree of 8 Bangladeshi SAC strains based on their single nucleotide polymorphism (SNP).

### 3.8. Phylogenomic analysis

A phylogenomic tree based on the whole genome was performed with closely related MDR *S. aureus* strains (Figure 8A). SAC 1, 4, 8, and 10 are in the same clade; in contrast, SAC 5 and 9 are in the same clade. Meanwhile, SAC 6 is in the clade with reference strains NCTC 8525.

Furthermore, the WGS-based phylogenomic tree (Figure 8A) places SAC 1 separately from the rest of the isolates, and it may have evolved from the same node as Dresden-275757 (isolated from Dresden, Germany), GR2 (isolated from Greece), and 11819-97 (isolated from Denmark). Besides, from the phylogenomic tree, it can be understood that strains originating from the same node (such as SAC 5 and SAC 9) and sharing a common ancestral origin exhibit very similar virulome and genotypic resistance patterns.

Through single nucleotide polymorphism (SNP) analysis, an SNP tree was generated (Figure 8B). The SNP tree supports the phylogenomic tree based on the WGS of the strains further: SAC 5 and 9 had a common ancestral origin, sharing a very close relationship reflected by their branch lengths. SAC 3, 4, 8, and 10 originate from the same root. However, unlike the whole-genome-based phylogenomic results, the SNP analysis shows a very close relationship between the strains SAC 1 and 6, grouping them in the same clade.

## 4. Conclusions

The study consisted of eight clinical MDR *S. aureus* strains collected from two hospitals based in Dhaka, Bangladesh. We identified several antibiotic-resistance genes, virulence determinants, toxin-antitoxin systems, and biosynthetic gene clusters in the studied strains through WGS analysis. Our study identified four different clonal complexes and the dominance of CC361, with four out of the eight strains belonging to ST361. Given the global prevalence of this sequence type, its rapid emergence in Bangladesh emphasizes the need for ongoing surveillance and research.

The glycopeptide vancomycin remains the gold-standard antibiotic for many clinical cases in Bangladesh for infections caused by MRSA. Our data from antibiotic resistance profiling is coherently suggestive of its efficacy, as 100% of the tested strains were found to be sensitive. Moreover, tigecycline and amikacin exhibited sufficient potency against the tested strains, showing potential as better therapeutic options to combat such infections. However, despite the current phenotypical sensitivity of the strains to vancomycin, tigecycline, and amikacin, data highlighted that their cautionary application is imperative, as the detection of relevant resistance genes raises concerns about the potential for the emergence of resistance under selective pressure. Such a situation also underscores the need to mitigate the reliance on phenotypic assays alone and incorporate more integrative approaches, including genotypic and phenotypic data, to guide treatment decisions. The detected wide array of genes conferring antibiotic resistance in *S. aureus* mediates their actions in many ways, including antibiotic efflux, inactivation, target alteration, target protection, and target replacement.

Notably, the almost-closed nature of the pan-genome reflected that the sequencing effort was sufficient and the WGS data have considerable practical utility. Further research should focus on identifying the most prevalent genes and their resultant proteins associated with resistance and pathogenicity of the species and target them for drug-designing experiments. Moreover, the studied strains allow for correlation with other clinical isolates of *S. aureus* in Bangladesh through phylogenomic analyses, enriching the data of *S. aureus* genome variability and helping shed light on its epidemiology in the country.

## Use of AI tools declaration

The authors declare they have not used Artificial Intelligence (AI) tools in the creation of this article.


