# Supplementary material for: Deciphering the genomic character of the multidrug-resistant Staphylococcus aureus from Dhaka, Bangladesh
Source: AIMS Microbiol. 2024 Sep 29;10(4):833–58. doi: 10.3934/microbiol.2024036 (PMC11609420; doi:10.3934/microbiol.2024036)
Supplement: Supplementary file 1 [file microbiol-10-04-036-s001.pdf]

AIMS Microbiology

AIMS Microbiology, 10(4): 833–858.
DOI: 10.3934/microbiol.2024036
Received: 05 April 2024
Revised: 02 September 2024
Accepted: 20 September 2024
Published: 29 September 2024

http://www.aimspress.com/journal/microbiology

Research article

Deciphering the genomic character of the multidrug-resistant *Staphylococcus aureus* from Dhaka, Bangladesh

Afia Anjum[†], Jarin Tabassum[†], Sohidul Islam, A. K. M. Imrul Hassan, Ishrat Jabeen and Sabbir R. Shuvo*

Department of Biochemistry & Microbiology, North South University, Dhaka, Bangladesh

* **Correspondence:** Email: sabbir.shuvo@northsouth.edu; Phone: 880255668200; Ext: 6263.

[†]These two authors contributed equally.

Table S1. Antibiogram result of 8 *S. aureus* strains.

Strains	Zone of Inhibition in Millimeters (mm)																	
	AMP	AMC	TZP	FOX	CAZ	CFM	CRO	CTX	FEP	ATM	MEM	AMK	GEN	CIP	LEV	ERY	TET	TGC	COL
SAC 1	0	13.67	13	0	0	0	0	0	0	0	23.67	21.33	21.67	8.67	13.67	0	26	22	0
SAC 3	0	15	17	15.33	0	0	11.33	0	0	0	19	19.67	22.67	10.33	19.67	0	0	20.67	0
SAC 4	0	13.33	17.33	15.33	0	0	0	0	0	0	20.67	16.67	22.33	10	13.67	0	26.67	21	0
SAC 5	0	13.67	17.67	12.67	0	0	0	0	0	0	18	20.33	24.33	27.33	26.33	26	27.67	23.33	0
SAC 6	0	8	11	0	0	0	0	0	0	0	0	23.67	10.67	11.67	16	0	25.33	23.33	0
SAC 8	0	9.67	13	11.67	0	0	0	0	0	0	0	17	20.67	11	14.33	0	25.33	20	0
SAC 9	0	10.67	12.33	9. 67	0	0	0	0	0	0	0	18.67	0	12	15.33	0	27	21	0
SAC 10	0	9.67	11.67	9. 67	0	0	0	0	0	0	0	18.67	23	9.67	15.67	0	27	22	0

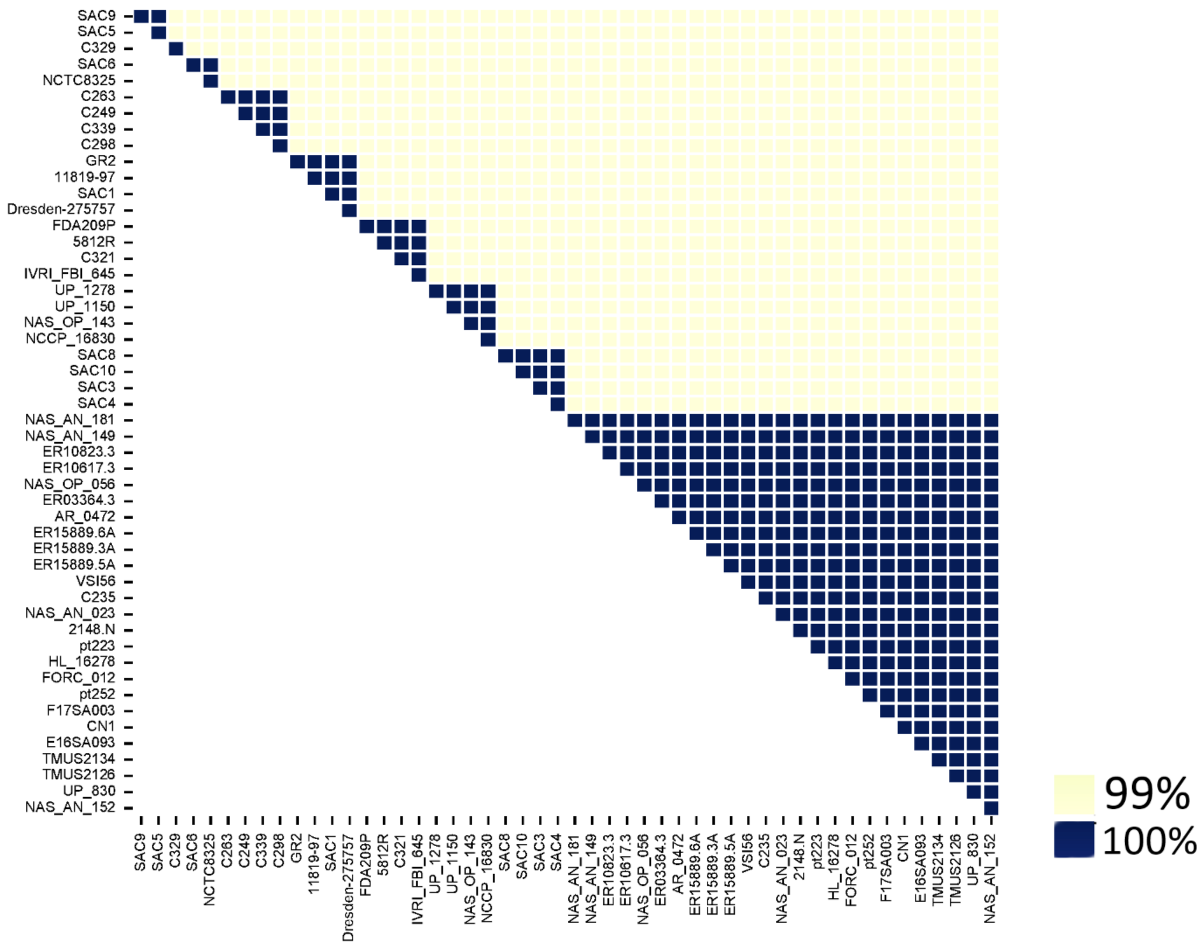

Figure S1. Matrix Heatmap of the 8 SAC strains alongside the 41 strains worldwide and reference strain NCTC8325 based on their Average Nucleotide Identity (ANI).

Table S2. The 50 *S. aureus* strains used in phylogenomic analysis.

Serial No.	NCBI identification number	Full Name	Short Name
1	JASXSJ000000000.1	*Staphylococcus aureus* strain SAC_01_SRS	SAC1
2	JASXSK000000000.1	*Staphylococcus aureus* strain SAC_03_SRS	SAC3
3	JASXSL000000000.1	*Staphylococcus aureus* strain SAC_04_SRS	SAC4
4	JASXSM000000000.1	*Staphylococcus aureus* strain SAC_05_SRS	SAC5
5	JASXSN000000000.1	*Staphylococcus aureus* strain SAC_06_SRS	SAC6
6	JASXSO000000000.1	*Staphylococcus aureus* strain SAC_08_SRS	SAC8
7	JASXSP000000000.1	*Staphylococcus aureus* strain SAC_09_SRS	SAC9
8	JASXSQ000000000.1	*Staphylococcus aureus* strain SAC_10_SRS	SAC10
9	CP016856.2	*Staphylococcus aureus* strain 2148.N chromosome	2148.N
10	CP123852.1	*Staphylococcus aureus* strain 5812R chromosome	5812R
11	CP003194.1	*Staphylococcus aureus* subsp. aureus 11819-97	11819-97
12	CP029649.1	*Staphylococcus aureus* strain AR_0472 chromosome	AR_0472

Continued on next page

Serial No.	NCBI identification number	Full Name	Short Name
13	CP127745.1	*Staphylococcus aureus* strain C235 chromosome	C235
14	CP127807.1	*Staphylococcus aureus* strain C249 chromosome	C249
15	CP127629.1	*Staphylococcus aureus* strain C263 chromosome	C263
16	CP127613.1	*Staphylococcus aureus* strain C298 chromosome	C298
17	CP127605.1	*Staphylococcus aureus* strain C321 chromosome	C321
18	CP127599.1	*Staphylococcus aureus* strain C329 chromosome	C329
19	CP127684.1	*Staphylococcus aureus* strain C339 chromosome	C339
20	CP003979.1	*Staphylococcus aureus* subsp. aureus CN1	CN1
21	CP054876.1	*Staphylococcus aureus* strain Dresden-275757 chromosome	Dresden-275757
22	CP031131.1	*Staphylococcus aureus* strain E16SA093 chromosome	E16SA093
23	CP030550.1	*Staphylococcus aureus* strain ER03364.3 chromosome	ER03364.3
24	CP051933.1	*Staphylococcus aureus* strain ER10617.3 chromosome	ER10617.3
25	CP051919.1	*Staphylococcus aureus* strain ER10823.3 chromosome	ER10823.3
26	CP075582.1	*Staphylococcus aureus* strain ER15889.3A chromosome	ER15889.3A
27	CP075581.1	*Staphylococcus aureus* strain ER15889.5A chromosome	ER15889.5A
28	CP075580.1	*Staphylococcus aureus* strain ER15889.6A chromosome	ER15889.6A
29	CP031130.1	*Staphylococcus aureus* strain F17SA003 chromosome	F17SA003
30	CP123853.1	*Staphylococcus aureus* strain FDA209P chromosome	FDA209P
31	CP010998.1	*Staphylococcus aureus* strain FORC_012	FORC_012
32	CP010402.1	*Staphylococcus aureus* subsp. aureus strain GR2	GR2
33	CP080564.1	*Staphylococcus aureus* strain HL16278 chromosome	HL16278
34	CP126185.1	*Staphylococcus aureus* strain IVRI_FBI_645 chromosome	IVRI_FBI_645
35	CP062419.1	*Staphylococcus aureus* strain NAS_AN_023 chromosome	NAS_AN_023
36	CP062416.1	*Staphylococcus aureus* strain NAS_AN_149 chromosome	NAS_AN_149
37	CP062362.1	*Staphylococcus aureus* strain NAS_AN_152 chromosome	NAS_AN_152
38	CP062412.1	*Staphylococcus aureus* strain NAS_AN_181 chromosome	NAS_AN_181
39	CP062402.1	*Staphylococcus aureus* strain NAS_OP_056 chromosome	NAS_OP_056
40	CP062399.1	*Staphylococcus aureus* strain NAS_OP_143 chromosome	NAS_OP_143
41	CP043843.1	*Staphylococcus aureus* strain NCCP 16830 chromosome	NCCP 16830
42	CP049496.1	*Staphylococcus aureus* strain pt223 chromosome	pt223
43	CP049441.1	*Staphylococcus aureus* strain pt252 chromosome	pt252
44	CP000253.1	*Staphylococcus aureus* subsp. aureus NCTC 8325	*S. aureus* NCTC 8325
45	AP014652.1	*Staphylococcus aureus* TMUS2126 DNA	TMUS2126

Continued on next page

Serial No.	NCBI identification number	Full Name	Short Name
46	AP014653.1	*Staphylococcus aureus* TMUS2134 DNA	TMUS2134
47	CP047790.1	*Staphylococcus aureus* strain UP_830 chromosome	UP_830
48	CP047786.1	*Staphylococcus aureus* strain UP_1150 chromosome	UP_1150
49	CP047822.1	*Staphylococcus aureus* strain UP_1278 chromosome	UP_1278
50	CP118044.1	*Staphylococcus aureus* strain VSI56 chromosome	VSI56

Table S3. Biofilm formation assay of 8 *S. aureus* strains**.** The biofilm formation was determined by using the following methods. ODcut = ODavg of negative control + 3 × standard deviation (SD) of OD of negative control; OD ≤ ODcut = non-biofilm former; ODcut < OD ≤ 2 × ODcut = weak-biofilm former; 2× ODcut < OD ≤ 4 × ODcut = moderate-biofilm former; OD > 4 × ODcut = strong-biofilm former [1].

	SAC 1	SAC 3	SAC 4	SAC 5	SAC 6	SAC 8	SAC 9	SAC 10	Blank
OD	0.275	0.21	0.184	0.225	0.202	0.226	0.193	0.189	0.206
	0.225	0.237	0.188	0.194	0.222	0.226	0.207	0.207	0.208
	0.239	0.245	0.188	0.209	0.219	0.219	0.184	0.176	0.179
Average	0.24633	0.230667	0.18667	0.20933	0.21433	0.223667	0.1947	0.19067	0.1977
Difference	0.0487	0.033	-0.011	0.0117	0.0167	0.026	-0	-0.007	
Biofilm former class	Weak	Weak	None	Weak	Weak	Weak	None	None	

Table S4. Mobilome identified in the studied *S. aureus* strains.

Strains	Number of Plasmids	ICE count	Number of Intact Prophage Regions
SAC 1	4	2 regions (Putative ICE with T4SS)	2
SAC 3	2	2 regions (Putative ICE with T4SS)	2
SAC 4	2	None	2
SAC 5	3	None	2
SAC 6	6	None	0
SAC 8	1	1 region (Putative ICE with T4SS)	1
SAC 9	3	None	2
SAC 10	4	2 regions (Putative ICE with T4SS)	1

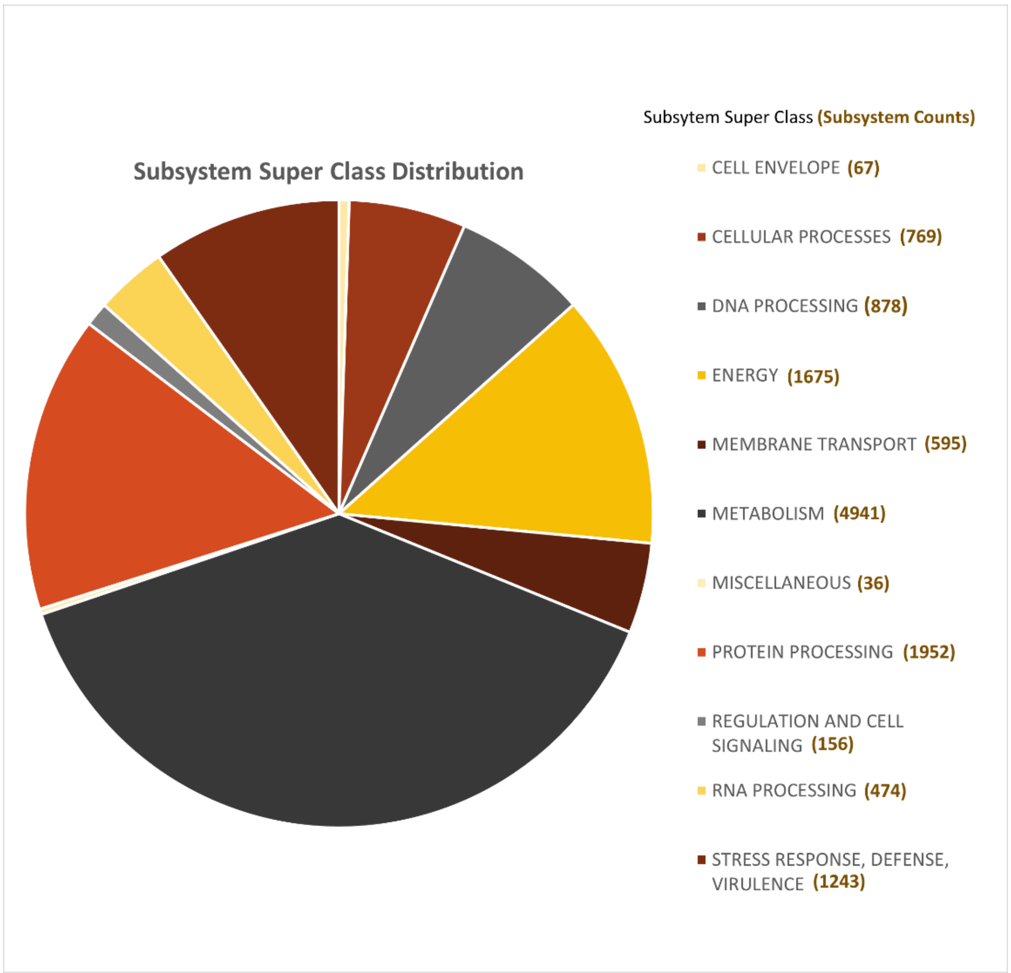

Figure S2. The subsystem super class distribution for all the strains. The number of subsystems is colored in brown.

Table S5. SCCmec predicted genes.

Strains	Predicted genes
SAC 1	subtype-IVa(2B); ccrA2; ccrB2
SAC 3	ccrC1-allele-2:1
SAC 4	ccrC1-allele-2:1
SAC 5	subtype-IVa(2B); ccrA2; ccrB2
SAC 6	subtype-IVc(2B); ccrA2; ccrB2
SAC 8	mec-class-C2; ccrC1-allele-2:1
SAC 9	subtype-IVa(2B); ccrA2; ccrB2
SAC 10	subtype-IVg(2B); ccrA2; ccrB2

Table S6. Identified Single Nucleotide Polymorphisms (SNPs) in the predicted antibiotic resistance genes.

Strain	Antibiotic Resistance Genes	SNPs in the Predicted Genes
SAC 1	*gyrA*	S84L
SAC 3	*gyrA*	S84L
SAC 4	*gyrA*	S84L
SAC 5	*gyrA*	S84L
SAC 8	*gyrA*; *parC*	S84L; S80F
SAC 9	*gyrA*	S84L
SAC 10	*gyrA*	S84L

References

1. Stepanović S, Vuković D, Dakić I, et al. (2000) A modified microtiter-plate test for quantification of staphylococcal biofilm formation. *J Microbiol Methods* 40: 175–179.

AIMS Press

© 2024 the Author(s), licensee AIMS Press. This is an open access article distributed under the terms of the Creative Commons Attribution License (http://creativecommons.org/licenses/by/4.0)
